# Functional diversity of BAI1 (ADGRB1): From angiostasis to synaptic remodeling and disease therapeutics

**DOI:** 10.1016/j.isci.2026.114656

**Published:** 2026-01-09

**Authors:** Aguo Li, Kenqi Zhang, Lei Tang, Xinyan Li, Xiaoye Huang, Jia Pan, Yijie Fang, Ping Xu, Jianhua Li, Hongyan Wang, Yong-Sheng Tu

**Affiliations:** 1 The Second Clinical College, Guangzhou Medical University, Guangzhou 511436, China; 2 School of Public Health, Guangzhou Medical University, Guangzhou 511436, China; 3 The Nanshan College, Guangzhou Medical University, Guangzhou 511436, China; 4 School of Pediatrics, Guangzhou Medical University, Guangzhou 511436, China; 5 The First Clinical College, Guangzhou Medical University, Guangzhou 511436, China; 6 Department of Pulmonary and Critical Care Medicine, Peking University Shenzhen Hospital, Shenzhen 518034, Guangdong, China; 7 State Key Laboratory of Respiratory Disease, Guangzhou Municipal and Guangdong Provincial Key Laboratory of Protein Modification and Disease, School of Basic Medical Sciences, Guangzhou Medical University, Guangzhou 511436, Guangdong, China; 8 Department of Pathology, School of Basic Medical Sciences, Guangzhou Medical University, Guangzhou 511436, China; 9 State Key Laboratory of Respiratory Disease, Guangzhou Municipal and Guangdong Provincial Key Laboratory of Protein Modification and Disease, Department of Physiology, School of Basic Medical Sciences, Guangzhou Medical University, Guangzhou 511436, China

**Keywords:** Health sciences, Natural sciences, Biological sciences, Biochemistry, Physiology

## Abstract

Brain-specific angiogenesis inhibitor 1 (BAI1/ADGRB1), a member of the adhesion G protein-coupled receptor (ADGR) family, is regulated at the transcriptional level through epigenetic mechanisms (e.g., MBD2, EZH2, and p53) and alternative promoters, resulting in molecularly distinct isoforms via differential promoters usage (e.g., intron 17-derived variants) and post-translational processing (e.g., matrix metalloproteinase 14 [MMP-14]-mediated Vstat40 release). In cellular and animal studies, both the full-length BAI1 and its proteolytically released extracellular domains have been showed to exhibit multifaceted bioactivity, including inhibition of angiogenesis, suppression of tumor progression, and modulation of immune responses via interactions with CD36, integrins, lipopolysaccharide (LPS), and phosphatidylserine (PtdSer). Furthermore, BAI1 plays crucial roles in neurodevelopmental processes such as synaptic plasticity, neuronal differentiation, and cellular debris clearance, with emerging links to neuropsychiatric disorders. Despite significant advances, critical gaps remain in understanding isoform-specific functions and activation mechanisms across different tissues. This review systematically integrates current knowledge on BAI1, focusing on its genomic regulatory mechanisms, structural isoform diversity, and multidimensional biological functions. It also underscores the need to explore the translational potential of BAI1 in oncology, neurodegenerative diseases, and immune dysregulation, which is essential for advancing our understanding of this complex receptor and its therapeutic applications.

## Graphical abstract

**Figure:**
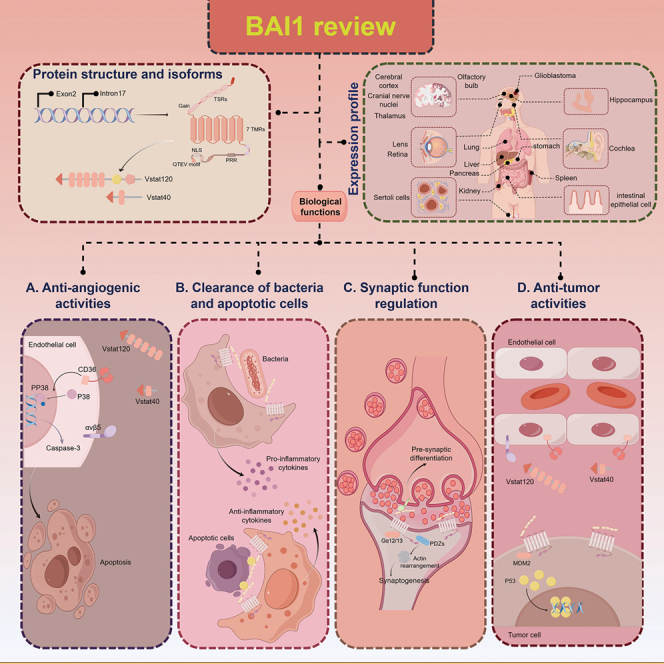

## Introduction

Brain-specific angiogenesis inhibitor 1(BAI1)/adhesion G protein-coupled receptor B1(ADGRB1), a member of the adhesion G protein-coupled receptor family, has emerged as a critical regulator of diverse physiological and pathological processes. Two other homologs of BAI1 with a similar overall architecture, named BAI2/ADGRB2 and BAI3/ADGRB3, belong to the same subfamily of ADGRs and have been identified in central nervous system (CNS).^1^^,^^2^ Initially identified as a p53-target gene encoding an anti-angiogenic factor in glioblastoma,^3^ BAI1 is now recognized for its multifaceted roles in tumor suppression, immune defense, synaptic plasticity, and tissue homeostasis. Its functional versatility stems from a unique multi-domain architecture—including thrombospondin type-1 repeats (TSRs), Arginine-Glycine-Aspartic acid (RGD) motifs, and adhesion G protein-coupled receptor autoproteolysis-inducing domain (GAIN). These structural features enable dynamic interactions with ligands such as phosphatidylserine (PtdSer), CD36, and integrins. These interactions trigger context-dependent signaling cascades, ranging from Rho/Rac-mediated cytoskeletal remodeling to ELMO/Dock180-dependent phagocytosis.^4^

Given the crucial roles of BAI1 in various physiological and pathological processes, understanding the origin and function of its diverse isoforms becomes essential. BAI1 biology is characterized by its ability to generate functionally distinct isoforms through alternative promoter usage, proteolytic cleavage, and post-translational modifications.^4^ For instance, cleavage by matrix metalloproteinases (MMPs) releases anti-angiogenic fragments (Vstat40),^5^ while membrane-bound C-terminal fragments represent a signaling-competent form of BAI1 protein and have been implicated in pathways potentially regulating synaptic development, such as Rho activation.^6^ Despite these advances, key questions remain unresolved: how do spatiotemporal expression patterns of BAI1 isoforms dictate their functional specificity in different tissues? and what mechanisms govern the crosstalk between BAI1-derived fragments?

These unresolved questions underscore the need for a comprehensive understanding of BAI1. This review aims to address three interconnected themes: molecular regulation of BAI1 expression and isoform diversity; mechanistic insights into BAI1’s roles in angiogenesis inhibition, apoptotic clearance, and synaptogenesis; and translational potential in diseases such as cancer, neurodegeneration, and inflammatory disorders. We advocate for isoform-specific investigations and targeted therapeutic strategies to exploit BAI1’s multifunctionality in precision medicine.

## BAI1 as an ADGR family member with multi-isoform complexity

### ADGRB1/Adgrb1 gene expression and regulation

The expression of ADGRB1 and its mouse ortholog Adgrb1 is tightly regulated by a complex network involving transcriptional factors, epigenetic modifications, and non-coding RNAs. The ADGRB1 gene (encoding the BAI1 protein in humans) and its mouse ortholog Adgrb1 are located on chromosome 8q24.3 (comprising 35 exons) and chromosome 15D3; 15 34.22 cM (containing 32 exons), respectively. ADGRB1 was initially isolated and identified as a novel p53-inducible gene containing at least one “functional” p53-binding site.^3^ However, subsequent studies have shown no consistent correlation between ADGRB1 mRNA levels and TP53 gene status in several glioma cell lines,^7^ suggesting that p53-mediated regulation of ADGRB1 may be cell type-specific. In glioblastoma, the cytosine-phosphate-guanine (CpG) island of exon 2 of the ADGRB1 gene is in a hypermethylated state,^8^ and methyl-CpG-binding domain protein 2 (MBD2) could selectively bind to the methylated CpG region and recruit histone deacetylase (HDAC), leading to a repressive chromatin environment, which hinders the binding of transcription factors to the ADGRB1 promoter.^8^^,^^9^ In addition, LncRNA embryonic stem cells expressed 1 (Lncenc1) promotes the enrichment of enhancer of Zeste homolog 2 (EZH2) on the Adgrb1 promoter, which facilitates the methylation modification of histone H3 lysine 27 (H3K27) of the Adgrb1 promoter, resulting in transcriptional repression of the Adgrb1 gene and subsequent downregulation of BAI1 expression.^10^ In neural stem cells (NSCs) and astrocytes, microRNA-325-3p binds to the 3′-untranslated region (3′UTR) of Adgrb1, inhibiting the expression of the Adgrb1 gene and playing an important regulatory role in the physiological and pathological processes such as proliferation, differentiation, and apoptosis of nerve cells.^11^ In addition, microRNA-3191-5p is also involved in the negative regulation of Adgrb1 gene.^12^

### Multiple transcripts and alternative promoter-driven isoform generation of ADGRB1/Adgrb1

**Figure 1:**
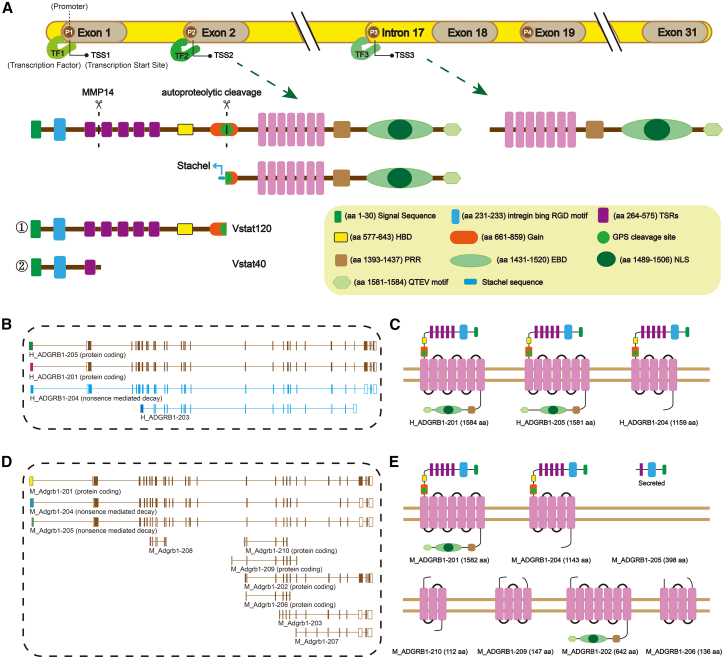
Structural and Transcriptomic Analysis of Human ADGRB1 and Mouse Adgrb1 Genes and Their Encoded Protein Isoforms. (A) Structure of membrane BAI1 protein generated from the complete mRNA sequence and alternative promoter transcript discovered by Parag et al.^4^ Compared to the membrane-bound CTF produced via autoproteolytic cleavage at the GPS site, the isoform generated from the alternative promoter transcripts lacks the GAIN domain and Stachel sequence; it may lack receptor activity that depends on conformational changes induced by the tethered agonist. (B and C) Known ADGRB1 (human) protein-coding transcripts (source: Ensembl genome browser, release 110). Each transcript has a unique first exon, indicated by a different color (B). The protein structure translated from the corresponding protein-coding transcript was predicted using Swiss-Model (https://swissmodel.expasy.org/) (C). (D and E) Known Adgrb1 (mouse) protein-coding transcripts (source: Ensembl genome browser, release 110). Each transcript has a unique first exon (D). The protein structure translated from the corresponding protein-coding transcript was predicted using Swiss-Model (E).

Previous studies have shown that alternative transcription initiation leads to the formation of transcripts with lengths different from that of the canonical ADGRB1 transcript, which is derived from the exon 2 or 5′ untranslated region (5′-UTR).^4^ Specifically, transcription can initiate from an alternative exon at the intron 17/exon 18 boundary (Figure 1A), and translation starting from a new start codon (ATG) or exon 19 start codons gives rise to two shorter isoforms (predicted molecular weights: 76.9/76.4 kDa and 70.8/70.5 kDa), as validated by immunoblot analysis. This transcriptional plasticity provides a mechanistic explanation for BAI1’s isoform diversity, although the physiological or pathological roles of these novel BAI1 isoforms in tissue homeostasis and disease remain unclear.^4^ Furthermore, Ensembl genome browser annotations reveal that both human ADGRB1 and mouse Adgrb1 encode multiple protein-coding transcripts, each featuring a unique first exon (Figures 1B–1E). However, the biological functions and cell/tissue-specific expression profiles of these isoforms remain largely unexplored, representing a frontier for understanding BAI1’s context-dependent activities.

### The BAI1 protein has a typical G protein-coupled receptor structure and generates isoforms through protein cleavage

The full-length BAI1 protein (predicted MW 173.5 kDa) is encoded by mRNA transcripts (NCBI: NM_001702.2/NM_174991; UniProt: O14514/Q3UHD1) and exhibits a modular architecture typical of ADGRs. The seven-helix transmembrane (7TM) domain promotes the oligomerization of BAI1,^13^^,^^14^ while the three intracellular loops (ICLs) mediate downstream signaling: ICL1 binds to the Murine Double minute 2 (MDM2) E3-ubiquitin ligase,^9^ and ICL3 binds to Gα12/13 that can activate the Rho signaling pathway.^6^ The ectodomain of BAI1 successively contains an N-terminal signal peptide for intracellular transport, an RGD integrin-binding motif, a predicted CUB domain,^15^ and five TSRs that can interact with PtdSer on apoptotic cells,^16^ lipopolysaccharide (LPS) on Gram-negative bacteria,^17^ Reticulon 4 receptor (RTN4R) on pre-synapses,^18^ and CD36 on endothelial cells.^19^ Adjacent to the TSRs, a hormone-binding domain (HBD) and a GAIN that triggers self-cleavage at the adjacent G protein-coupled receptor proteolysis site (GPS) are located.^20^ The intracellular C-terminal tail of BAI1 has a proline-rich region (PRR) that interacts with IRSp53 (also known as BAP2),^21^^,^^22^ an ELMO/DOCK-binding domain (EBD) that recruits ELMO/DOCK180 and activates the Rac1 signaling pathway,^17^ and a terminal amino acid QTEV (glutamine-threonine-glutamic acid-valine) domain that serves as an interaction site for PDZ domain-containing proteins such as the scaffold proteins MAGI-3, PSD-95, BAI-associated protein 1 (BAP1), and Tiam1/Par3 that potentiate Rac1 activation.^6^^,^^23^^,^^24^ This region also contains a nuclear localization sequence (NLS), suggesting potential non-canonical functions^25^ (Figure 1A).

The complexity of BAI1 isoforms, as evidenced by western blot analysis of brain and other organs, indicates the presence of numerous tissue-specific variants.^4^ Proteolytic cleavage is an important source of diversity. The full-length BAI1 protein undergoes two well-characterized proteolytic events. First, cleavage by MMP-14 at the site between TSR1 and TSR2 produces an approximately 40 kDa anti-angiogenic N-terminal fragment (NTF) of BAI1 (Vstat40), which contains the RGD motif and the first TSR.^5^ Second, autoproteolytic cleavage at the GPS site can generate a low-abundance 120 kDa fragment (angiostatin-120/Vstat120) encompassing all five TSRs.^26^ These TSR-containing fragments exhibit independent anti-angiogenic activities,^5^^,^^26^ while full-length BAI1 mediates PtdSer-dependent apoptotic cell clearance by macrophages and glia via its TSR motifs.^16^^,^^27^ Notably, although alternative promoter-derived short isoforms contain specific motifs, whether they can independently recapitulate full-length or secreted fragment functions remains undefined—highlighting a critical gap in isoform-specific functional characterization.

## BAI1 exhibits specific expression and distribution patterns

The expression profile of BAI1 protein is both highly tissue-specific and cell-type-dependent. In the human brain, BAI1 expression is the highest in the white and deep gray matter of the cerebral cortex, particularly in the dendrites of neurons.^7^^,^^28^ In mice, BAI1 is also highly expressed in multiple brain regions, including the cerebral cortex and hippocampus, as well as the cranial nerve nuclei and the astrocytes of the olfactory bulb.^27^ In the hippocampus, abundant BAI1 expression is closely related to learning and memory processes.^29^ In contrast, the expression of BAI1 is relatively low in regions such as the thalamus.^30^

Beyond the CNS, BAI1 plays critical roles in sensory systems. In the eye, BAI1 is mainly distributed in the lens and retina. In the lens, BAI1 is expressed in epithelial cells and fiber cells, participating in the development of the lens and maintaining its transparency.^31^ In the retina, BAI1 is enriched in the outer and inner plexiform layers and is expressed in various cell types, including retinal ganglion cells, bipolar cells, and photoreceptor cells, potentially involved in retinal development, signal transduction, and neuroprotection.^27^ In the auditory system, BAI1 is expressed in the spiral ganglion neurons (SGNs) of the cochlea and is enriched in the postsynaptic density (PSD) of inner hair cells (IHCs), which assists in the proper localization of α-amino-3-hydroxy-5-methyl-4-isoxazolepropionic acid (AMPA) receptors (GluR2-4) on the PSD and is involved in maintaining normal hearing.^32^ Furthermore, BAI1 is detected in multiple peripheral tissues such as the lungs,^33^ pancreas,^34^ colon,^35^ stomach^36^ and kidneys,^37^ with varying abundances.

At the cellular level, BAI1 is widely present in multiple cell types. In the human brain, immunohistochemical studies have found that the BAI1 protein is mainly distributed in the cytoplasm and membranes of neuron cells, especially being prominently expressed in pyramidal cells of the cerebral cortex.^28^ BAI1 is also present in neurons of regions such as the cerebellum and hippocampus, suggesting its potential involvement in neural activities and information transmission in these areas.^27^ Meanwhile, BAI1 is expressed in astrocytes. Although its expression level is relatively low, it participates in the remodeling of the extracellular matrix and the regulation of neuroinflammation during the processes of neuroinflammation and injury repair.^27^ Additionally, BAI1 is a marker of Myo/Nog cells, which are present in the skin, eyes, and brain and play a key role in embryonic development and tissue repair.^31^^,^^38^^,^^39^ Myo/Nog cells are activated in proliferative vitreoretinopathy (PVR), proliferating and differentiating into myofibroblasts that express α-smooth muscle actin (α-SMA) and striated muscle myosin. These cells generate contractile forces leading to retinal detachment. Intravitreal injection of cytotoxic agents specifically targeting BAI1 to eliminate Myo/Nog cells may emerge as a novel therapeutic strategy for PVR.^38^ In microglia, the resident macrophages of the CNS, BAI1expression is closely related to the phagocytic clearance of apoptotic cells. Immunostaining with specific antibodies reveals that BAI1 is mainly distributed in the cytoplasm and processes of microglia, and upregulated during the phagocytosis of apoptotic cells.^40^ However, its expression in peripheral macrophages remains debated. While some studies, using methods such as gene expression analysis and protein detection, have failed to detect BAI1 protein or transcript in monocytes and macrophages under standard conditions,^41^ some studies have found that under certain specific conditions, such as bacterial infection, macrophages BAI1 expression may be induced and it may participate in immune responses and inflammation regulation.^17^ This discrepancy highlights the necessity for further investigation to clarify the expression pattern and function of BAI1 in macrophages.

## Specific motifs of BAI1 determine its biological functions

The specific motif structure within the BAI1 molecule is the key to understanding its diverse biological functions. These structural modules confer precise ligand-binding capabilities, which trigger distinct signaling cascades. These pathways, in turn, direct cellular behaviors that are fundamental to maintaining physiological homeostasis or contributing to disease pathogenesis.

### Secreted BAI1 fragments have anti-angiogenic activities

**Figure 2:**
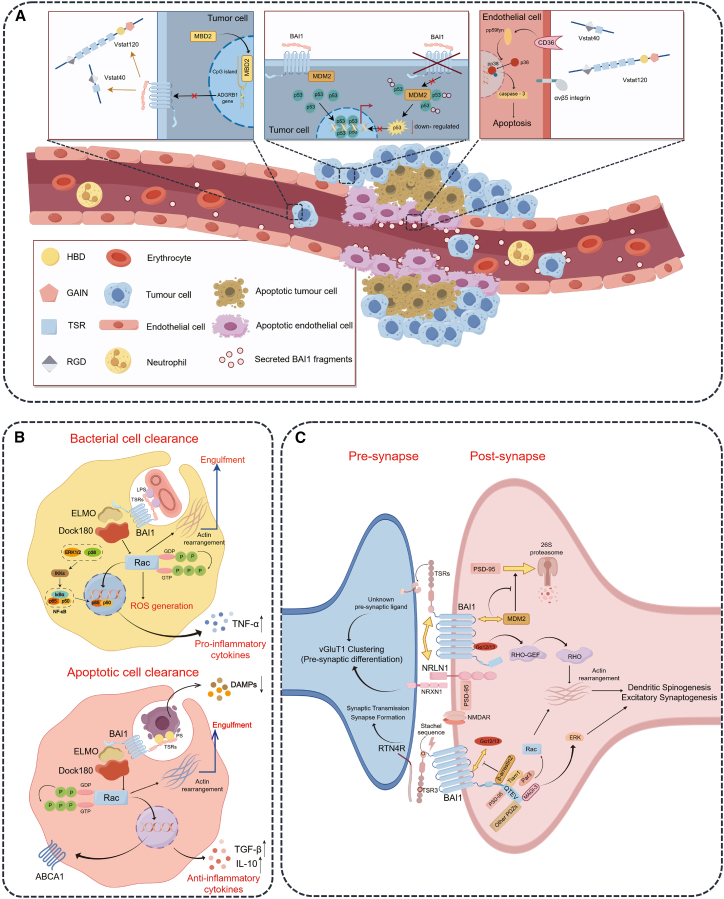
Schematic illustration systematically demonstrating the multifunctional roles of BAI1 across diverse biological processes (A) Vstat40, Vstat120, and BAI1-TSR inhibit angiogenesis by engaging CD36 and integrins on endothelial cells, triggering apoptosis. Concurrently, BAI1 stabilizes p53 by inhibiting MDM2, and its transcription is epigenetically suppressed by MBD2. (B) BAI1 facilitates apoptotic cell clearance by recognizing PtdSer and acts as a pattern recognition receptor for Gram-negative bacteria by binding LPS through its TSRs. (C) BAI1 regulates synaptogenesis and synapse development through *trans*-synaptic signaling. The figure was rendered by FigDraw.

BAI1 is a transmembrane protein with anti-angiogenic activity, and its mechanism of action depends on the core fragments with anti-proliferative effects produced by its cleavage.^42^^,^^43^ Currently identified core fragments include the Vasculostatin 120 fragment (Vstat120, about 120 kDa)^26^ and the Vstat40 fragment (about 40 kDa).^5^ When BAI1 was first cloned, it was recognized that its TSRs might have anti-angiogenic potential similar to those of the known anti-angiogenic protein TSP-1.^44^ Researchers such as Van Meir^26^ proposed the existence of a soluble extracellular region of BAI1 called Vasculostatin/Vstat120, and similar extracellular fragments of BAI1 were detected in the conditioned medium of 293T cells transfected with BAI1 and brain lysates. Exogenous expression of Vstat120 inhibited tumor growth in a dose-dependent manner. Its anti-angiogenic activity requires the participation of CD36, which activates the caspase-3-mediated apoptosis pathway of endothelial cells^45^ (Figure 2A). The histidine-rich glycoprotein (HRGP) can competitively bind to Vstat120 through its CLESH domain, thereby extracellularly reversing the inhibitory effect of Vstat120 on endothelial cell migration and angiogenesis,^45^ which further confirmed the anti-angiogenic and anti-tumor activities of Vstat120 and its specific mechanism. Similarly, Vstat40 also has the activity of inhibiting the migration of CD36^+^ endothelial cells (Figure 2A), the formation of vascular structures, and angiogenesis *in vivo* depending on the interaction between its TSR and CD36 ^5^. In addition, another study suggested the probable existence of a ∼30 kDa extracellular fragment containing the BAI1 TSRs, but its precise identity and origin were not clearly verified.^42^^,^^43^ The authors further proposed that this fragment had anti-angiogenic effects through blockade of αvβ5 integrin signaling,^42^ the RGD motif present in BAI1 may contribute to this process as it is a common integrin-binding motif.^46^

The potent anti-angiogenic properties of the secreted BAI1 fragments position them as promising therapeutic candidates for ophthalmic diseases related to abnormal angiogenesis. In diabetic retinopathy (DR) and neovascular age-related macular degeneration (nAMD), the abnormally generated blood vessels, which are triggered primarily by VEGF signaling, compromise retinal integrity and threaten vision.^47^^,^^48^ Current anti-VEGF therapies, though effective, underscore the clinical importance of angiogenesis inhibition. Notably, it has been demonstrated^43^ that subconjunctival injection of the ADGRB1 gene in a rabbit corneal neovascularization model achieved potent and sustained anti-angiogenic effects comparable to those of anti-VEGF antibodies, with no significant toxicity. Given this efficacy and BAI1’s native role in vascular regulation, intravitreal delivery of BAI1 fragments represents a compelling translational strategy worthy of further investigation.

### BAI1-mediated bacterial recognition and phagocytosis

BAI1 plays a crucial role in macrophages-mediated defense against bacterial infections. In mouse mononuclear macrophages (J774 macrophages) and bone-marrow-derived macrophages (BMDMs), BAI1 preferentially recognize a series of Gram-negative bacterial pathogens, such as *Salmonella typhimurium*, *Escherichia coli*, and *Campylobacter jejuni*.^17^^,^^49^^,^^50^ This recognition is mediated by direct interaction between the TSRs in the BAI1 ectodomain and the core oligosaccharide of LPS.^17^

Following bacterial binding, BAI1 binds to ELMO1 (engulfment cell motility 1) through its cytoplasmic α-helix region, triggering the activation of the Rho-family GTPase Rac1 and coordinating the formation of membranous pseudopods to drive bacterial internalization^17^ (Figure 2B). Whether in phagocytic cells (such as J774 macrophages, BMDMs) or non-phagocytic heterologous cells (such as CHO cells), BAI1 overexpression enhances bacterial internalization; conversely, impairment of BAI1 function reduces the bacterial internalization efficiency.^17^ In addition, BAI1-mediated recognition and internalization of bacteria triggers a pro-inflammatory response: it synergizes with TLR4 in LPS-induced pro-inflammatory signal transduction and induces the release of the key pro-inflammatory cytokine tumor necrosis factor-α (TNF-α) via activation of the NF-κB and MAP kinase pathways, which is driven by the downstream effector ELMO1.^17^^,^^50^ Moreover, beyond its role in bacterial internalization, Rac1 is an essential component of the NADPH oxidase complex. In macrophages, when BAI1 recognizes Gram-negative bacteria, activated Rac1 promotes the assembly of the cytoplasmic regulatory subunit with the transmembrane catalytic subunit gp91phox, thereby activating NADPH oxidase and triggering the production of reactive oxygen species (ROS).^49^ The BAI1-mediated bacterial uptake and ROS production together enhance the bactericidal activity of macrophages against Gram-negative bacteria.

### BAI1-mediated recognition and phagocytosis of apoptotic cells

BAI1 has a highly specific recognition ability and can accurately recognize PtdSer exposed on the surface of apoptotic cells. BAI1 specifically binds to PtdSer through the TSRs in its extracellular region, and this binding process is the initial step in initiating the clearance of apoptotic cells. After recognizing apoptotic cells, BAI1 engages the ELMO/Dock180 complex to activate the small GTPase Rac and then lead to the rearrangement of the actin cytoskeleton, promoting the formation and extension of pseudopods and the formation of phagocytic vesicles (Figure 2B). The phagocytic vesicles gradually enclose the apoptotic cells to form phagosomes, which is an important intermediate link in the clearance of apoptotic cells.^16^ In addition, the BAI1 receptor has also been proven to activate the Rho pathway through a Gα12/13-dependent mechanism, thereby promoting cytoskeleton reorganization.^6^ In contrast to the induction of pro-inflammatory molecules usually triggered by bacterial internalization, BAI1 signaling during apoptotic cell clearance establishes an immunologically silent resolution by preventing secondary necrosis and consequent liberation of damage-associated molecular patterns (DAMPs), such as HMGB1, IL-1, and IL-33,^51^ and concurrently promoting active secretion of key anti-inflammatory mediators, predominantly TGF-β.^52^

BAI1 participates in the clearance process of apoptotic cells in multiple cell types, demonstrating its extensive biological functions. Osteoclasts,^53^ microglia,^40^ and macrophages,^54^ which are professional mononuclear phagocytes, originate from the common monocyte lineage and all have functional expression of phagocytic receptors including BAI1. Osteoclasts are in a special microenvironment in bone tissue, where BAI1 may play a potential role in mediating the clearance of residues of apoptotic cells, mediating the apoptosis of osteoclasts in the late phase of osteoclastogenesis and maintaining the stability of the bone tissue microenvironment.^53^^,^^55^ In microglia, after knocking down BAI1, the formation of phagosomes is significantly slower, and the process from the initial contact with apoptotic neurons to the formation of phagocytic cups is prolonged. Although apoptotic cells can be finally tightly enclosed, the delay in the formation speed reflects the important regulatory role of BAI1 in the early stage of phagosome formation.^40^ BAI1-expressing astrocytes frequently contain phagocytosed apoptotic debris, with BAI1 accumulating in the phagocytic cups during the phagocytosis process, which is strong evidence for BAI1-mediated phagocytosis of apoptotic cells by astrocytes.^27^ In the normal physiological environment *in vivo*, macrophages rely on BAI1 to accurately recognize apoptotic cells, thereby initiating the subsequent phagocytosis process and maintaining the homeostasis of tissue cells. Generally, macrophages take up a large amount of cholesterol during the process of clearing apoptotic cells. In the development of diseases such as atherosclerosis, there is an imbalance in lipid metabolism. Cholesterol accumulates in macrophages to form foam cells, promoting plaque formation. Some studies have shown that BAI1 is related to the up-regulation of ATP-binding cassette transporter (ABCA1), a key protein for cholesterol efflux in macrophages. Therefore, the BAI1-related signaling pathway triggered by apoptotic cells may be involved in regulating lipid metabolism and reducing the accumulation of lipid-rich macrophages in tissues.^54^

In addition, BAI1 also plays a role in non-professional phagocytes such as intestinal epithelial cells and Sertoli cells in the testis. Research by Lee et al.^52^ showed that during intestinal inflammation, specific overexpression of BAI1 in colonic epithelial cells enhances their ability to clear apoptotic cells, which reduces the accumulation of such cells in intestinal tissues with a concomitant decrease in the release of pro-inflammatory factors such as TNF and IL-16, thereby contributing to maintain the normal structure and function of intestinal tissues. The accumulation of apoptotic cells also affects the homeostasis of testicular tissues and spermatogenesis. Sertoli cells can effectively clear these apoptotic cells through BAI1-mediated phagocytosis, avoiding the accumulation of apoptotic cells in the testis. This helps to maintain the balance of the testicular cell population and ensure the normal development and maturation of germ cells.^56^ In addition, the expression of BAI1 in Sertoli cells can compensate for the deficiency of MerTK (one of the PtdSer receptors) and rescue the phagocytic defect of Sertoli cells caused by the reduction of MerTK,^57^ indicating that PtdSer receptors do not exist in isolation but form a network with other molecules involved in apoptotic cell clearance in the cell.

In-depth research on the mechanism of BAI1-mediated apoptotic cell clearance not only helps to better understand the regulatory process of apoptosis but also provides new ideas and targets for the treatment of diseases related to abnormal apoptotic cell clearance, such as infertility, Alzheimer’s disease, systemic lupus erythematosus, rheumatoid arthritis, and atherosclerosis.

### BAI1 functions related to the nervous system

Genetic and clinical evidence implicate BAI1 in brain development and function. Variants in ADGRB1 gene are found in autism spectrum disorder (ASD) patients. Some ASD patients have epilepsy, brain structural changes, and developmental delays.^58^ Mice lacking full-length BAI1 show various social deficits, increased epilepsy susceptibility, and abnormal brain development, which are clinically relevant phenotypes.^59^ Also, BAI1 expression is markedly downregulated in Parkinson’s disease (PD) patients’ substantia nigra tissue and MPTP/MPP^+^-induced PD models, triggering neuronal death.^60^ Understanding the multidimensional biological functions of BAI1 in the nervous system is crucial for developing targeted multi-target intervention strategies, and the enrichment of BAI1 in the PSD provides a structural basis for its function at the synapse.^6^

#### Participating in the regulation of synapse formation and maturation

BAI1 can activate the Rho signaling pathway through a Gα12/13-dependent pathway. The activated Rho signaling pathway further regulates the dynamic changes of the cytoskeleton, creating favorable conditions for synapse formation and maturation^6^ (Figure 2C). It is worth noting that the N-terminal and 7TM regions of BAI1 remain physically associated after cleavage at the GPS motif, and this association occludes the Stachel sequence that functions as a tethered agonist located between GAIN and 7TM regions,^61^ maintaining the receptor in an inactive state. When the NTF binds to its ligand and dissociates from the CTF, the Stachel sequence is exposed, thereby activating G protein-dependent signaling.^6^^,^^20^^,^^62^ In addition, the C-terminal of BAI1 can bind to the PDZ domains of multiple synaptic proteins. On the one hand, it interacts with the PDZ domain of MAGI-3, enhancing ERK activation and helping neurons establish more effective synaptic connections.^6^ On the other hand, it regulates Rac1 activation and actin assembly by recruiting the Par3/Tiam1 polarity complex to synaptic sites via the PDZ-binding motif of BAI1, thereby regulating excitatory synapse development.^23^ At the same time, BAI1 interacts with β-arrestin2 independently of the PDZ motif, which can terminate or weaken BAI1-mediated G-protein-dependent signaling to avoid cell damage caused by excessive signal activation.^6^ Notably, this PDZ-dependent recruitment of Par3/Tiam1 represents a neuron-specific adaptation of Rac-activating capability mediated by BAI1 protein, contrasting with its ELMO/Dock180-mediated Rac activation through the helical domain during phagocytosis. This modular signaling strategy enables a single receptor to transduce diverse extracellular cues into context-appropriate cytoskeletal rearrangements, supporting functions ranging from phagocytosis to synaptogenesis. BAI1 also promotes excitatory synaptogenesis by coordinating bidirectional *trans*-synaptic signaling. In terms of forward signaling, BAI1 can activate itself through its N-terminal Stachel sequence, then leads to the activation of Rac1 and subsequent synaptogenesis, a process that is inhibited in neurons with BAI1 knockdown.^62^ In the reverse signaling aspect, BAI1 can also induce the aggregation of the presynaptic protein vGluT1 through *trans*-synaptic signaling, thereby promoting presynaptic differentiation.^62^ Additionally, BAI1 co-localizes with the synapse-promoting cell adhesion molecule neuroligin-1 (NLGN1) at the postsynaptic site, and there is a functional dependency between the two. NLGN1 aids in the synaptic localization of BAI1, while the NTF of BAI1 binds to NLGN1 and promotes dendritic spine and synapse growth that depends on NLGN1.^62^ Research has found that BAI1 has a high-affinity binding capability with members of the RTN4R family including RTN4R, RTN4RL1, and RTN4RL2. This binding depends on the interaction between the TSR3 of BAI1 and the leucine-rich repeat (LRR) of the RTN4 receptor. This interaction involves unique glycosylation modifications, such as *O*-fucosylation at Thr424 and C-mannosylation at Trp418 and Trp415, which enhance the binding affinity of BAI1 with the RTN4R. BAI1 within neurons binds to RTN4R to regulate dendritic branching and synapse formation, while BAI1 expressed by glial cells binds to neuronal RTN4R to inhibit excessive axon extension. This differential binding pattern allows BAI1 to regulate its functions at different stages of neuronal development. For example, in the early stage, BAI1 expressed by glial cells inhibits excessive axon extension to avoid redundant connections, and in the later stage, the BAI1-RTN4R interaction between neurons promotes synapse stability and inhibits excessive dendritic branching.^18^ In the late stage of neuronal development, BAI1 can also activate the GTPase activity of RhoA by interacting with the Rho-GTPase regulatory protein Bcr, thereby inhibiting excessive dendritic growth.^63^ This process is crucial for maintaining the morphological and functional stability of neurons, and its abnormality may lead to neurodevelopmental disorders or neurodegenerative diseases.

#### Regulating synaptic plasticity

In terms of synaptic plasticity, BAI1 affects synaptic function by regulating the stability of PSD-95. BAI1 interacts with the E3 ubiquitin ligase MDM2, thereby sequestering it and preventing the ubiquitination and degradation of its substrate, PSD-95 (Figure 2C). This mechanism was validated in Adgrb1 gene-knockout (KO) mice. These mice showed severe spatial learning and memory impairments, accompanied by enhanced long-term potentiation (LTP) and impaired long-term depression (LTD) in hippocampal CA1 neurons, as well as a significant thinning of the PSD thickness.^29^

#### Potential impacts on neurodevelopment and neural circuit disorders

The functions of BAI1 in the nervous system are also reflected in its potential impacts on neurodevelopment and neuropsychiatric diseases. The ADGRB1 gene is located in a hotspot region prone to *de novo* mutations in patients with ASD.^64^ Moreover, BAI1 protein expression is upregulated in mouse models of Rett syndrome and methyl-CpG-binding protein 2 (MeCP2) duplication syndrome. These findings suggest that BAI1 may play an important role in neurodevelopment and neuropsychiatric diseases.^29^ In the auditory system, BAI1 protein is expressed in cochlear SGNs and enriched in the PSD of IHCs. BAI1 is crucial for the aggregation of AMPA receptors (GluR2-4) in the PSD of SGNs. In Adgrb1-deficient mice, although the functions of IHCs are normal, SGNs cannot effectively transmit sound information, resulting in a significant increase in the hearing threshold. This indicates that BAI1 plays a key role in auditory signal transmission, and its functional deficiency may lead to hearing impairment.^32^

In summary, these findings nominate BAI1 as a compelling therapeutic target for neural disorders. Further elucidation of BAI1 signaling, regulation, and activation by ligands will likely lead to substantial new insights into neurological disorders that have a basis in aberrant synapse formation and morphology.

### A key promoter in cell fusion

Beyond its roles in phagocytosis and angiogenesis, BAI1 emerges as a facilitator of cell fusion processes. While disruption of Adgrb1 or antibody blockade of BAI1 alone minimally impacts fertilization, simultaneous inhibition of BAI1 and CD36, or co-deletion of Adgrb1 and MerTK, drastically reduces *in vitro* fertilization rates. This indicates that BAI1 mediates sperm-egg fusion in coordination with other PtdSer receptors.^65^ During the process of myoblast fusion, PtdSer exposed on apoptotic myoblasts acts as a key signaling molecule to interact with the TSRs of normal myoblast BAI1 and activate the ELMO/Dock180/Rac1 signaling pathway, thereby promoting myoblast fusion.^66^ In addition, during the early fusion of osteoclasts, stimulation with M-CSF and RANKL induces the non-apoptotic externalization of PtdSer on the surface of osteoclast precursor cells. BAI1 recognizes and binds to these externalized PtdSer, enhancing the adhesion between osteoclast precursor cells, promoting close cell-to-cell contact, and facilitating cell fusion.^55^

In summary, the structural features of BAI1 closely determine its multiple important functions. It has a long extracellular N-terminal region rich in various adhesion domains, such as TSRs, RGD, and HBDs. These domains endow BAI1 with the ability to interact with the extracellular matrix, other proteins, and ligands, thus participating in processes such as cell adhesion, migration, and signal transduction. The G-protein-coupled domain and the seven-transmembrane structure enable it to couple with G proteins and activate downstream signaling pathways, such as the Rho and ERK pathways, thereby regulating cytoskeleton reorganization, synapse formation, and neurotransmitter transmission. The α-helix and PDZ-binding motifs in the intracellular C-terminal region are closely related to the formation of cell pseudopods, synapse occurrence, and function regulation. Therefore, in-depth research on the structure and function of BAI1 is of great significance for understanding normal physiological functions and disease mechanisms. We have summarized the interacting proteins of BAI1, the specific motifs they participate in, and the biological functions they mediate in current research into a list, as shown in Table 1.

**Table 1:** Comprehensive list of interacting partners identified for BAI1

Isoforms		NT interaction	Region	Function	Reference
Full-length BAI1 (∼170 kDa)		1. lipopolysaccharides	1. TSRs	1. bacterial internalization	Das et al.^17^
		2. PtdSer	2. TSRs	2. recognize apoptotic cells and Myoblast fusion	Park et al., Sokolowski et al., Elliott et al., Hochreiter-Hufford et al.^16^^,^^27^^,^^56^^,^^66^
		3.MMP-14	3.Ser326 Leu327	3. cleave BAI1 NT	Cork et al.^5^
		4. G8 mAb	4.TSR3	4. cell recognition, tracking, sorting, and analysis	Gerhart et al.^39^
		5.RTN4R-LRR region	5. TSR3	5. synaptogenesis	Wang et al.^18^
		6. ATP11A	6. NTF	6. reduce G protein-dependent signaling activity	Lala et al.^14^
		7. NRLN1	7. NTF	7. synaptogenesis	Tu et al.^62^
		8. unknown	8.Stachel sequence	8. synaptogenesis	Tu et al.^62^
Secreted fragment	BAI1-TSR (∼30 kDa)	αvβ5 Integrin	unknown	block proliferation of endothelial cells	Koh et al., Yoon et al.^42^^,^^43^
	Vstat120 (∼120 kDa)	1. CD36	1. TSRs	1. inhibit angiogenesis	Kaur et al.^26^
		2. Integrin	2. RGD	2. block proliferation of endothelial cells	Kaur et al.^26^
		3. HRGP (CLESH domain)	3. TSRs	3. reverse the inhibitory effect of Vstat120 on endothelial cell migration and angiogenesis	Klenotic et al.^45^
	Vstat40 (∼40 kDa)	CD36	TSR1	inhibit angiogenesis	Cork et al.^5^

Isoforms		CT interaction	Region	Function	Ref.

Full-length BAI1 (∼170 kDa)		1. ELMO1	1. α-Helix region	1. bacterial internalization, apoptotic cell clearance, and the expression of ABCA1	Park et al., Das et al., Fond et al.^16^^,^^17^^,^^54^
		2. ELMO2	2. α-Helix region	2. myoblast fusion	Hochreiter-Hufford et al.^66^
		3. Tiam1	3. PDZ-binding motif	3. synaptogenesis	Duman et al.^23^
		4. Par3	4. PDZ-binding motif	4. synaptogenesis	Duman et al.^23^
		5. Gα12/13	5. ICL3	5. activate the Rho pathway	Stephenson et al.^6^
		6. PSD-95, MAGI-3, MAGI-1, MAGI-2, INADL, SAP97, Chapsyn-110, MALS-1, Densin-180, and PAPIN 1	6. PDZ-binding motif	6. synaptic function regulation	Stephenson et al., Zhu et al.^6^^,^^29^
		7. MDM2	7. ICL1	7. synaptic function regulation, stabilization of p53 protein levels, and suppression of tumor progression.	Zhu et al.^29^
		8. BAP1	8. PDZ-binding motif	8. unknown	Shiratsuchi et al.^24^
		9. BAP3	9. CTF	9. unknown	Shiratsuchi et al.^67^
		10. BAP2 (or IRSp53)	10. PRR	10. unknown	Oda et al.^22^
		11. BAP4	11. aa 1254–1516	11. unknown	Koh et al.^68^

## BAI1 plays a dual role in the occurrence and development of tumors

BAI1 has been established as a pivotal tumor suppressor, with its inactivation contributing to malignant progression across multiple cancer types. Originally identified as a p53-target gene mediating anti-angiogenic and anti-tumor effects in brain tissue,^3^ BAI1 is frequently lost or significantly downregulated in glioblastoma (GBM) tissues in contrast to the characteristic expression pattern in normal brain tissue.^69^ And in astrocytomas, BAI1 expression levels demonstrate a graded decline with increasing pathological grade.^70^ Mechanistically, promoter hypermethylation of the ADGRB1 gene facilitates MBD2-dependent transcriptional repression, functionally silencing BAI1 expression in GBM.^8^ Restoration of BAI1 expression in glioma models significantly inhibits glioma cell proliferation and reduces tumor vascular density,^69^ indicating the protein’s direct anti-tumor activity, which reveals that therapeutic strategies targeting MBD2 may achieve tumor control by alleviating BAI1 transcriptional repression.^8^ BAI1 also exerts tumor-suppressive functions through regulation of the p53 signaling pathway as demonstrated in a mouse medulloblastoma (MB) model, where BAI1 competitively binds to the E3 ubiquitin ligase MDM2, thereby shielding p53 from ubiquitin-mediated degradation and enhancing its antitumor activity.^9^ Independently, BAI1’s anti-tumor effects involve its proteolytic fragments. The engineered oncolytic viral vector (e.g., rapid antiangiogenesis mediated by oncolytic virus) expressing Vstat120 successfully suppresses tumor angiogenesis and intracranial growth in GBM models,^19^^,^^71^ and another cleavage product, Vstat40, also exhibits anti-angiogenic properties *in vitro* and in animal studies, though its direct anti-tumor efficacy warrants further validation.^72^

BAI1’s tumor-suppressive function extends beyond CNS malignancies, with emerging evidence revealing distinct mechanistic pathways across diverse cancer types. In lung cancer models, BAI1 orchestrates metabolic reprogramming through coordinated upregulation of stearoyl-CoA desaturase 1 (SCD1) and suppression of 3-hydroxy-3-methylglutaryl-CoA reductase (HMGCR), effectively inhibiting the Warburg effect and reducing tumor cell energy acquisition.^33^ Similarly, in mouse models of intrahepatic cholangiocarcinoma (ICC), the expression and function of Adgrb1 gene are regulated by circular RNA (circRNA) CircUGP2 through a dual mechanism: direct interaction with the transcription factor PURB to promote Adgrb1 transcription and concurrent sequestration of miR-3191-5p to stabilize Adgrb1 transcripts, which activate the Adgrb1/p53 signaling pathway, thereby suppressing the proliferation, migration, and invasion of ICC cells.^12^ The clinical relevance of BAI1 is further underscored by its correlation with improved disease-free survival in breast cancer, where extracellular fragment Vstat120 delivered via oncolytic viral vectors can inhibit angiogenesis in Nestin-high tumor cells.^73^ Complementary evidence from bladder cancer demonstrates an inverse relationship between BAI1 expression and both tumor aggressiveness and VEGF levels, potentially mediated through the inhibition of MMP-9-mediated VEGF release.^74^ This anti-angiogenic role is further corroborated in colorectal cancer, where reduced BAI1 expression significantly associates with enhanced vascular invasion, distant metastasis, and tumor stromal vascularization,^75^ further supporting its role as a microenvironmental angiogenesis inhibitor.

Notably, recent studies have uncovered paradoxical roles of BAI1 in specific tumor types. In non-smoking non-small cell lung cancer (NSCLC) patients, elevated nuclear BAI1 expression correlates with reduced disease-specific survival.^76^ Similar observations in renal cell carcinoma report significantly shorter overall survival (OS) and progression-free interval (PFI) in patients with high BAI1 expression.^37^ These findings imply tissue-specific regulatory networks governing BAI1 function, underscoring the necessity of contextualizing its clinical utility as a tumor biomarker.

In summary, BAI1 participates in tumor regulation through multidimensional mechanisms. Its anti-angiogenic activity, p53 stabilization, and metabolic modulation position it as a promising therapeutic target. However, its functional heterogeneity across tumor types highlights the need to unravel its molecular regulatory networks, thereby advancing precision oncology.

## Conclusion

To date, enormous strides have been made in unveiling BAI1 as a multifunctional hub in vascular biology, immunology, and neuroscience, significantly advancing our understanding of ADGRs. Intriguingly, non-canonical BAI1 isoforms derived from alternative promoters have recently emerged. However, critical questions concerning their action mechanism remain unanswered. One of the most pressing questions is whether these isoforms exhibit signaling capacities like serving as a docking station for PDZ-containing proteins analogous to the CTF of BAI1, since they possess identical C-terminal functional motifs. Future studies employing isoform-specific overexpression and knockdown models combined with *in vitro* and *in vivo* functional assays are essential. This review also integrates an overview of the diverse functions of BAI1, which mediates apoptotic cell clearance and recognition of Gram-negative bacteria in maintaining tissue homeostasis, and regulates dendritic branching, synaptogenesis, and synaptic plasticity in the nervous system. Additionally, secreted BAI1 fragments demonstrate therapeutic promise in ocular diseases and tumor. To determine the therapeutic utility of the BAI1 protein, identification of all BAI1 ligands that bind to the large NTF and CTF and deeper structure-based studies of BAI1 are required, as structure determines function. The site- and cell-specific variation of BAI1 activity establishes it as a versatile regulatory protein with wide potential for biomedical applications, while existing controversies regarding its expression and distribution patterns should be resolved. By addressing these gaps, BAI1 may unlock novel strategies for treating a diverse range of human diseases.

## Acknowledgments

This work was supported by the 2023 Guangdong Provincial Department of Education Featured Innovation Projects for General Higher Education Institutions in Guangdong Province (no. 2023KTSCX105), the Research Capacity Enhancement Project of Guangzhou Medical University in 2023, the Student Innovation Capacity Enhancement Program Project of Guangzhou Medical University in 2022, the Special Funds for Science and Technology Innovation Strategy of Guangdong Province (no. pdjh2021b0411), Guangdong Basic and Applied Basic Research Foundation (grant no. 2021A1515012145), and the Science and Technology Project of Guangzhou (grant no. 202102080324).

## Author contributions

A.L. sorted out the literature, and designed and created table and figures. A.L., K.Z., L.T., X.L., X.H., J.P., and Y.F. sorted out the literature and wrote the manuscript. P.X., J.L., H.W., and Y.-S.T. were responsible for conceptualization, funding acquisition, writing the original draft, and revising the manuscript. All authors have reviewed and approved the final version of the manuscript.

## Declaration of interests

The authors declare no competing interests.
